# The Effects of Testosterone on Hypothalamic and Serum Oxytocin Levels Are Affected by the Estrogen Milieu in Female Rats

**DOI:** 10.3390/nu16152533

**Published:** 2024-08-02

**Authors:** Moeka Arata, Kou Tamura, Hidenori Aoki, Hiroki Noguchi, Asuka Takeda, Saki Minato, Shota Yamamoto, Riyo Kinouchi, Kanako Yoshida, Yuri Yamamoto, Takashi Kaji, Takeshi Iwasa

**Affiliations:** 1 Department of Obstetrics and Gynecology, Institute of Biomedical Sciences, Tokushima University Graduate School, 3-18-15 Kuramoto-cho, Tokushima 770-8503, Japan; arata.moeka@tokushima-u.ac.jp (M.A.);; 2 Department of Renal and Genitourinary Surgery, Graduate School of Medicine, Hokkaido University, Sapporo 060-0808, Japan

**Keywords:** oxytocin, estrogen, androgen

## Abstract

Previous studies have suggested that the effects of androgens on body weight (BW) and appetite are affected by the estrogen milieu in females; however, the mechanism underlying these effects remains unclear. We hypothesized that androgens may affect endogenous oxytocin (OT), which is a hypothalamic anorectic factor, and that these effects of androgens may be altered by the estrogen milieu in females. To investigate this hypothesis, in the present study, we examined the effects of testosterone on peripheral and central OT levels in ovariectomized female rats that did or did not receive estradiol supplementation. Ovariectomized female rats were randomly divided into non-estradiol-supplemented or estradiol-supplemented groups, and half of the rats in each group were concurrently supplemented with testosterone (i.e., rats were divided into four groups, *n* = 7 per each group). We also measured peripheral and central OT receptor (OTR) gene expression levels. As a result, we found that testosterone increased serum and hypothalamic OT levels and OT receptor mRNA levels in non-estradiol-supplemented rats, whereas it had no effects on these factors in estradiol-supplemented rats. In addition, testosterone reduced food intake, BW gain, and fat weight in non-estradiol-supplemented rats, whereas it did not have any effects on BW, appetite, or fat weight in estradiol-supplemented rats. These findings indicate that the effects of androgens on OT may be affected by the estrogen milieu, and elevated OT levels may be related to the blunting of appetite and prevention of obesity under estrogen-deficient conditions.

## 1. Introduction

Estrogen, a sex steroid hormone, has a variety of beneficial effects in women and is also involved in the maintenance of nutrient metabolism, i.e., it plays an important role in the regulation of food intake (FI) and preventing excessive body weight (BW) gain in women [1,2,3]. On the other hand, the roles of androgens in FI, energy metabolism, and BW regulation in women have not been fully investigated. Although several studies have indicated that androgens increase FI and fat weight in women of reproductive age [4,5,6,7], their effects in other women, e.g., post-menopausal women, remain unclear. Interestingly, it has been shown that androgens have favorable effects on metabolic maintenance in men. For example, testosterone, a representative androgen, has been suggested to play an important role in the prevention of excess weight gain and obesity. Thus, we hypothesized that the effects of androgens on female nutrient metabolism may depend on the estrogen milieu, i.e., they may have unfavorable effects in the presence of estrogen and favorable effects or less effectiveness in estrogen-deficient conditions. To further investigate this hypothesis, we compared the effects of testosterone on BW regulation in ovariectomized (OVX) rats that did or did not receive estradiol supplementation. As a result, we found that testosterone promoted weight gain and obesity under estradiol-supplemented conditions but had inhibitory effects on weight gain and obesity under non-estrogen-supplemented conditions. These results support our hypothesis [1]. However, the neuroendocrine mechanisms underlying these estrogen-dependent differences in the effects of testosterone could not be determined in our experiments.

To examine these mechanisms, we focused on oxytocin (OT), which is a hormone involved in metabolism that is affected by sex steroid hormones. OT is an amino acid neuropeptide that is produced in the paraventricular nucleus of the hypothalamus and the supraoptic nucleus. It acts on G-protein-coupled OT receptors (OTRs) and influences several behaviors, including social behavior, reproduction, and lactation [8]. Recently, it has been revealed that OT plays an important role in BW and appetite regulation. OTR-deficient mice exhibited late-onset obesity accompanied by increased amounts of abdominal fat and impaired cold-induced thermogenesis [9]. In addition, the administration of OT has been shown to ameliorate metabolic and nutritional disorders, causing reductions in BW, appetite, and fat mass in many species, including humans [10,11,12,13,14,15,16,17]. These results indicate that both endogenous and exogenous OT have favorable effects on BW regulation and prevent obesity and adiposity in many species. Furthermore, our previous studies indicated that the effects of OT are affected by the gonadal steroid milieu, and that the effects of the gonadal steroid milieu on appetite and BW regulation may be mediated by OT [18,19,20,21]. Namely, hypothalamic and peripheral levels of OT in OVX rats were lower than those seen in OVX rats that received estradiol supplementation [20]. Similarly, serum OT levels in women of reproductive age were increased under hyperestrogenic conditions [21]. On the other hand, peripheral OT levels in female rats that received androgen supplementation were lower than those seen in intact female rats [10,18]. The administration of exogenous OT decreased FI, BW, and fat weight in the former (androgen-supplemented) rats [10,18]. In diseases such as PCOS, hyperandrogenism is known to increase the risk of obesity and nutritional metabolic diseases before menopause. In contrast, it has been suggested that these risks are reduced after menopause. In the present study, we hypothesized that androgens prevent overeating and obesity by increasing OT levels in postmenopausal women. On the other hand, it is hypothesized that these mechanisms are not invoked in the premenopausal period, making the risk of obesity and nutritional metabolic diseases more likely.

Based on our results, we hypothesized that the effects of testosterone on endogenous OT levels in females may be altered by the estrogen milieu, and that such diverse effects may be related to the abovementioned differences in the effects of testosterone on BW and appetite observed in our previous study. To investigate this hypothesis, in this study, we examined OT levels, FI, BW gain, and body fat in OVX female rats that received chronic testosterone administration with or without estradiol supplementation. In addition, peripheral and central OTR levels were also measured. This is because the mRNA expression levels of the OTR are greatly affected by female gonadal steroid hormones [21].

## 2. Materials and Methods

### 2.1. Animals

Eight-week-old Wistar female adult rats (200–230 g) were purchased from Charles River Laboratories Japan, Inc. (Kanagawa, Japan), and kept in a room under controlled light (12 h light, 12 h darkness; lights turned on at 08:00 and turned off at 20:00) and temperature (24 °C) conditions, with food and water provided ad libitum. In total, 28 rats were used in this study. All animal experiments were carried out according to the ethical standards of the institutional animal care and use committee of the University of Tokushima. Ovariectomy, tube implantation, and decapitation were carried out as in our previous study [1]. At 9 weeks of age, rats were ovariectomized bilaterally and housed individually after the surgery. The data and samples from these animals were already used for our previous study, and part of the data about body composition and samples were randomly selected for our present study. Thus, part of the results presented in this study, i.e., data about body composition, were included in our previous study. On the other hand, the main outcomes, i.e., OT, OTR, and hormone levels, were newly measured for our present study and not published previously.

### 2.2. Effects of Chronic Testosterone Administration on Ovariectomized Rats That Did Not Receive Estradiol Supplementation (OVX)

Four weeks after undergoing an ovariectomy (at 13 weeks of age), the rats were randomly assigned to a testosterone-treated (testosterone) or untreated (control) group (*n* = 7 per group). In the testosterone group, a silastic tube filled with crystalline testosterone (inner diameter: 3 mm, outer diameter: 5 mm, length of the filled part: 30 mm) (As One Co., Ltd., Tokyo, Japan) was implanted into each rat (De Vries et al., 1994 [22]). In the control group, an empty tube was implanted into each rat. At 16 days after the implantation procedure, the rats were sacrificed by decapitation after their BW and cumulative FI had been measured. The brain, blood, visceral fat (parametrial, perirenal, and mesenteric deposits), and subcutaneous fat (the inguinal deposit) were collected. The weights of the visceral fat and subcutaneous fat were measured, and tissue samples (around 300–400 mm^3^) of the visceral (parametrial) and subcutaneous fat were dissected out. Serum and tissue samples were stored, and other tissue samples of visceral and subcutaneous fat were fixed as in our previous study [1].

### 2.3. Effects of Chronic Testosterone Administration on Ovariectomized Rats That Received Estradiol Supplementation (OVX + E2)

Four weeks after undergoing an ovariectomy (at 13 weeks of age), all of the rats were implanted with a silastic tube filled with crystalline estradiol (length of the filled part: 3 mm) (Le et al., 2014 [23]). At the same time, the rats were assigned to either the testosterone or control group, as described above (*n* = 7 per group). At 16 days after the implantation procedure, the rats were sacrificed by decapitation and the weight of each collected tissue was measured, and then samples were collected and stored or fixed, as described above.

### 2.4. Hormone Assay

Whole blood was centrifuged at 3000 rpm for 20 min at 4 °C, and the serum was sent to a commercial laboratory (SRL, Tokyo, Japan), where its OT levels were measured using a chemiluminescent enzyme immunoassay (ASKA Pharma Medical Co., Ltd., Kanagawa, Japan). The limit of detection for serum OT was 15 pg/mL.

### 2.5. Quantitative Real-Time Polymerase Chain Reaction

Whole hypothalamic explants were dissected from frozen brains, as described previously [18]. Total RNA was isolated and then cDNA was synthesized. PCR analysis was performed, and the mRNA expression levels of OT and the OTR were quantified. The mRNA expression levels were normalized to that of a housekeeping gene.

### 2.6. Statistical Analyses

All results are expressed as the means ± standard error of the mean (SEM). A Student’s *t*-test for data with equal variances or a Mann–Whitney U test for data with high variance were used for statistical analyses. *p*-values < 0.05 were considered to be significant. Cohen’s d (small effect = 0.2, medium effect = 0.5, large effect = 0.8) and r (small effect = 0.1, medium effect = 0.3, large effect = 0.5) are reported when analyses were undertaken by a Student’s *t*-test and Mann–Whitney U test, respectively. Spearman’s rank correlation coefficient was used for correlation analysis.

## 3. Results

### 3.1. Effects of Chronic Testosterone Administration on BW and FI in Ovariectomized Rats That Did (OVX + E2) or Did Not (OVX) Receive Estradiol Supplementation

**Figure 1:**
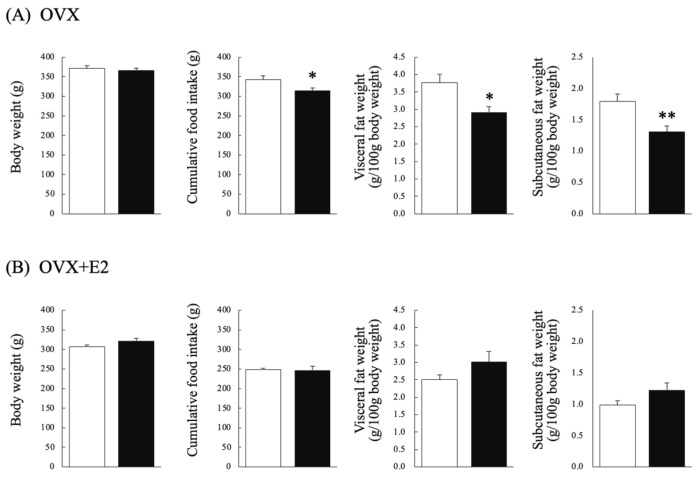
Body weight (BW), cumulative food intake (FI), visceral fat weight (g/100 g BW), and subcutaneous fat weight (g/100 g BW) in control (⧠) and testosterone-administered (■) female rats. The upper and lower rows show the results for ovariectomized rats that (**A**) did not (OVX) and (**B**) did (OVX + E2) receive estrogen supplementation, respectively. *n* = 7 per group. Data are expressed as means ± SEM. * *p* < 0.05, ** *p* < 0.01.

In the OVX group, there were no significant differences in BW after implantation between the testosterone and control groups. Cumulative FI after implantation was significantly lower in the testosterone group than in the control group (Mann–Whitney U test: *p* = 0.048, U = 9, r = 0.53) (Figure 1A). In the OVX + E2 group, there were no significant differences in BW after implantation between the testosterone and control groups. Similarly, there were no significant differences in cumulative FI after implantation between the testosterone and control groups (Figure 1B).

### 3.2. Effects of Chronic Testosterone Administration on Visceral Fat Weight and Subcutaneous Fat Weight in Ovariectomized Rats That Did (OVX + E2) or Did Not (OVX) Receive Estradiol Supplementation 

In the OVX group, visceral fat weight (g/100 g BW) was significantly lower in the testosterone group than in the control group (Mann–Whitney U test: *p* = 0.011; U = 5, r = 0.67). Similarly, subcutaneous fat weight (g/100 g BW) was significantly lower in the testosterone group than in the control group (Student’s *t*-test: *p* < 0.01; df = 12, t = 3.301, d = 1.60) (Figure 1A). In the OVX + E2 group, there were no significant differences in visceral fat weight (g/100 g BW) between the testosterone and control groups (Figure 1B).

### 3.3. Effects of Chronic Testosterone Administration on Serum OT Levels in Ovariectomized Rats That Did (OVX + E2) or Did Not (OVX) Receive Estradiol Supplementation 

**Figure 2:**
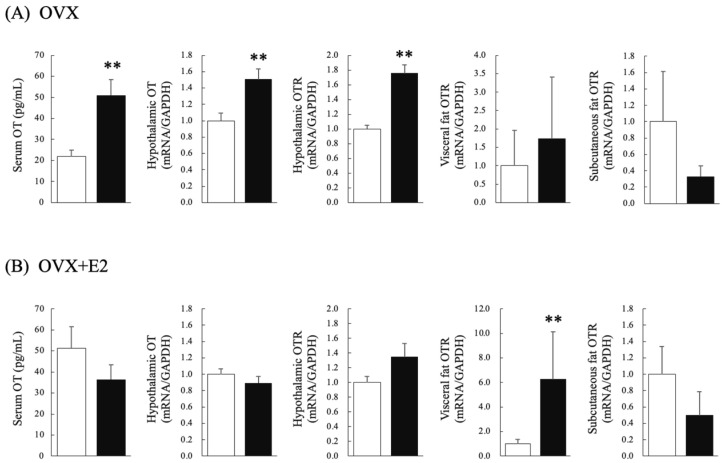
Serum OT concentrations, hypothalamic mRNA levels of OT and the OTR, and visceral and subcutaneous fat OTR mRNA levels in control (⧠) and testosterone-administered (■) female rats. The upper and lower rows show the results for ovariectomized rats that (**A**) did not (OVX) and (**B**) did (OVX + E2) receive estrogen supplementation, respectively. *n* = 7 per group. Data are expressed as means ± SEM. ** *p* < 0.01.

In the OVX group, the testosterone group exhibited significantly higher serum OT levels than the control group (Student’s *t*-test: *p* =0.004; df = 12, t = −3.607, d = 1.93) (Figure 2A). In the OVX + E2 group, there were no significant differences in serum OT levels between the testosterone and control groups (Figure 2B).

### 3.4. Effects of Chronic Testosterone Administration on Hypothalamic OT mRNA Levels, and Hypothalamic, Visceral Fat, and Subcutaneous Fat OTR mRNA Levels in Ovariectomized Rats That Did (OVX + E2) or Did Not (OVX) Receive Estradiol Supplementation 

In the OVX group, hypothalamic mRNA levels of OT and the OTR were significantly higher in the testosterone group than in the control group (Student’s *t*-test: hypothalamic OT: *p* < 0.01, df = 12, t = −3.137, d = 5.30; Mann–Whitney U test: hypothalamic OTR: *p* < 0.01, U = 0, r = 0.84) (Figure 2A). There were no significant differences in OTR mRNA levels in visceral or subcutaneous fat between the testosterone and control groups. In the OVX + E2 group, there were no significant differences in hypothalamic mRNA levels of OT and the OTR between the testosterone and control groups (Figure 2B). The testosterone group had significantly higher visceral fat OTR mRNA levels than the control group (Mann–Whitney U test: *p* = 0.000, U = 17, r = 0.15). Subcutaneous fat OTR mRNA levels of the testosterone and control groups did not differ significantly.

### 3.5. Correlations between Serum Testosterone Levels and Serum or Hypothalamic OT Levels in Ovariectomized Rats That Did (OVX + E2) or Did Not (OVX) Receive Estradiol Supplementation

**Figure 3:**
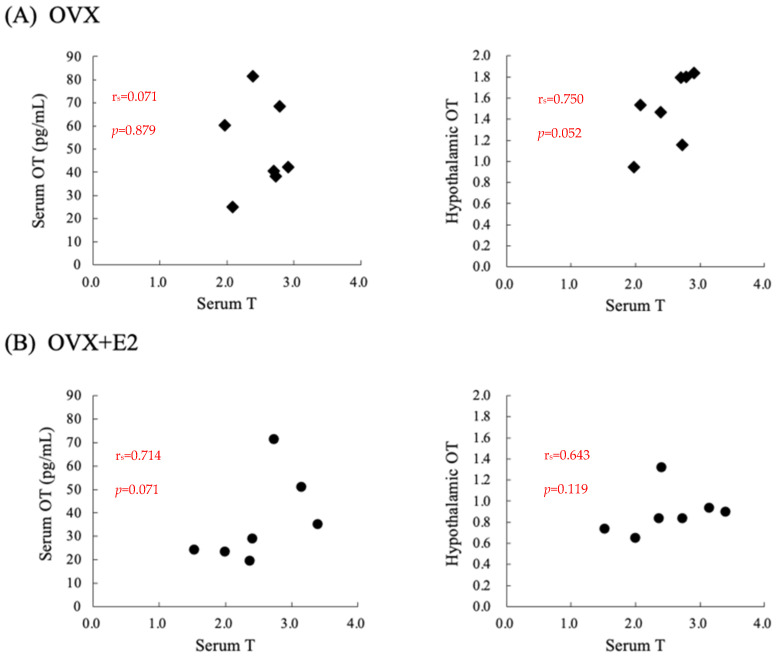
Correlations between serum testosterone levels and serum OT or hypothalamic OT mRNA levels. The upper and lower rows show the results for ovariectomized rats that (**A**) did not (OVX) and (**B**) did (OVX + E2) receive estrogen supplementation, respectively.

In the OVX group, there was no correlation between serum testosterone and OT levels, or between serum testosterone levels and hypothalamic OT mRNA levels (Figure 3A). In the OVX + E2 group, there was no correlation between serum testosterone and OT levels, or between serum testosterone levels and hypothalamic OT mRNA levels (Figure 3B).

## 4. Discussion

Energy balance is closely related to reproductive function in most species. Estrogen and androgens (sex steroid hormones) are heavily involved in the regulation of FI, energy metabolism, and BW in mammals and humans [7]. Regarding estrogen, estrogen deficiency due to ovariectomy increases FI and BW in female animals, but these effects can be prevented by estradiol replacement, indicating that estrogen plays an important role in preventing hyperphagia and excess BW gain in females [7,24,25]. On the other hand, the roles of androgens in the regulation of FI, energy metabolism, and BW in females have not been fully evaluated; however, some studies have indicated that androgens may increase FI and promote adiposity in female animals and women of reproductive age [4,5,6,7], indicating that androgens may have effects that promote weight gain and obesity [7,24,25]. Interestingly, these effects of androgens on energy balance may be altered by the estrogen milieu, i.e., androgens increase FI, BW, and fat weight in the presence of estrogen, whereas they reduce these parameters in estrogen-deficient conditions [1]. However, the neuroendocrine mechanisms underlying the contradictory estrogen-dependent effects of androgens could not be determined in our previous studies.

In a previous study, we suggested that central and peripheral levels of OT, which are related to energy balance, are influenced by estrogen; namely, hypothalamic OT mRNA and serum OT levels are increased by estrogen in OVX female rats [20]. Similar results have been observed in human studies, i.e., serum OT levels were found to be increased under high estrogen conditions in women receiving ovarian stimulation [21]. Furthermore, it has been shown that estrogen increases OT levels via estrogen receptors (ERα) on OT-producing neurons [26,27,28]. Thus, we hypothesized that androgens may also affect central and peripheral OT levels, and these effects of androgens on OT may be altered by the estrogen milieu. In this study, we found that testosterone did not have any effects on serum OT or hypothalamic OT or OTR mRNA levels under estrogen-supplemented conditions, whereas it increased both serum and hypothalamic levels of these factors under non-supplemented conditions. In addition, testosterone did not affect BW, FI, or visceral fat weight under estrogen-supplemented conditions. These findings indicate that the effects of androgens on OT may be affected by the estrogen milieu, and elevated OT levels may be related to the prevention of hyperphagia and obesity under estrogen-deficient conditions. Similar results were obtained in our previous study, in which the effects of testosterone on OT levels were evaluated in male rats, i.e., we found that testosterone increased serum OT and hypothalamic OTR mRNA levels and decreased body fat weight in male rats after orchidectomy [18]. The latter study supported our previous findings that androgens increase OT levels under estrogen-deficient conditions.

In the present study, testosterone increased visceral fat OTR mRNA levels under estrogen-supplemented conditions, whereas it did not affect visceral fat OTR mRNA levels under non-estrogen-supplemented conditions. These results regarding visceral fat levels of the OTR contradicted those concerning serum OT, hypothalamic OT, and OTR levels. Although we cannot confirm the reasons for this discrepancy, the following mechanisms might be related: Namely, testosterone may primarily act to increase OTR mRNA levels in visceral fat as seen in estrogen-supplemented conditions. However, testosterone may predominantly act to increase hypothalamic OT/OTR mRNA and serum OT levels in non-estrogen supplemented conditions, consequently, reducing visceral fat weight. In this condition, OTR mRNA levels in visceral fat may not be changed as one of the counter-regulations to prevent too much reduction in weight. 

The limitations of this study are as follows: We did not find any correlation between serum testosterone levels and serum OT or hypothalamic OT mRNA levels in estrogen-supplemented or non-estrogen-supplemented conditions. We supposed that OT levels may increase when serum testosterone levels increase above a threshold level, but they may plateau if testosterone levels increase further. Future studies about the relationship between serum testosterone levels and central and peripheral OT/OTR levels should be investigated. Nonetheless, this is the first study to examine changes in the effects of testosterone on endogenous OT levels in female rats in the presence or absence of estrogen.

Also, comparisons are made between the OVX and OVX + E2 groups for this experiment. The OVX group is comparable to the OVX/E2 group as it has had ovariectomies and is, therefore, not affected by endogenous E2. In the present experiment, OT and OT receptor levels were examined to investigate the relationship between hormones and feeding factors, without focusing on the hypothalamus. Vasopressin V2 receptor (V2R) and arginine vasopressin (AVP) mRNA levels were not examined. Future research will examine the relationship with the hypothalamus.

## 5. Conclusions

In summary, testosterone increased OT levels and decreased FI, BW gain, and fat weight under estrogen-deficient conditions, whereas it did not affect OT levels or the aforementioned parameters under estrogen-supplemented conditions. Until now, the effects of androgens on metabolic functions in females and the mechanisms underlying these effects have remained unclear. The present study suggests that OT is a key factor in the pathophysiological roles of androgens in female metabolism.

## Data Availability

Data are contained within the article.
